# Inoculum Size and False-Positive Detection of NDM- and OXA-48-Type Carbapenemases Using Two Multiplex Lateral Flow Assays

**DOI:** 10.3390/diagnostics14121274

**Published:** 2024-06-17

**Authors:** Chung-Ho Lee, Huiluo Cao, Shuo Jiang, Tammy Ting-Yan Wong, Cindy Wing-Sze Tse, Pak-Leung Ho

**Affiliations:** 1 Department of Clinical Pathology, Kwong Wah Hospital, Hospital Authority, Hong Kong, China; lch101@ha.org.hk (C.-H.L.);; 2 Department of Microbiology, Queen Mary Hospital, University of Hong Kong, Hospital Authority, Pokfulam Road, Pokfulam, Hong Kong, China; 3 Carol Yu Centre for Infection, University of Hong Kong, Hong Kong, China

**Keywords:** carbapenemase, lateral flow assay, clinical microbiology, Enterobacterales

## Abstract

The NG-Test CARBA 5 and Carbapenem-resistant K.N.I.V.O. Detection K-Set are lateral flow assays (LFAs) that rapidly detect five carbapenemases (KPC, NDM, IMP, VIM and OXA-48-like). We evaluated the effect of inoculum size on the performance of these two assays using 27 Enterobacterales isolates. Whole-genome sequencing (WGS) was used as the reference method. Using the NG-Test CARBA 5, eight *Serratia* spp. and six *M. morganii* isolates showed false-positive NDM results with a high inoculum. Using the Carbapenem-resistant K.N.I.V.O. Detection K-Set, eight *M. morganii*, four *Serratia* spp. and one *K. pneumoniae* isolates showed false-positive NDM and/or OXA-48-like bands at large inoculum sizes, while the other two *M. morganii* isolates demonstrated false-positive NDM and OXA-48-like results at all inoculum sizes. The false-positive bands varied in intensity. WGS confirmed that no carbapenemase gene was present. No protein sequence with a ≥50% identity to NDM or OXA-48-like enzymes was found. This study emphasizes the importance of assessing inoculum size in the diagnostic evaluation of LFAs.

## 1. Introduction

Carbapenems are broad-spectrum antibiotics commonly used to treat infections caused by multi-drug resistant organisms such as extended-spectrum β-lactamase (ESBL)-producing Enterobacterales. However, the emergence of carbapenem-resistant Enterobacterales, first reported in the 1980s, has significantly undermined the effectiveness of this class of antibiotics and poses a major threat to patient care [1]. Carbapenem-resistant Enterobacterales can be divided into carbapenemase-producing Enterobacterales (CPE) and non-carbapenemase-producing Enterobacterales [2]. CPE are of particular concern due to the rapid spread of carbapenemase genes through horizontal gene transfer, contributing to both community and nosocomial outbreaks [3,4]. The early detection of CPE in clinical and screening specimens is critical for guiding appropriate antimicrobial therapy and implementing infection control measures.

The detection of CPE can be achieved through phenotypic and genotypic methods. Phenotypic methods include culture-based assays (e.g., the carbapenem inactivation test, modified Hodge test), colorimetric assays (e.g., the Carba-NP test), immunologic assays and matrix-assisted laser desorption/ionization time-of-flight mass spectrometry (MALDI-ToF MS). Genotypic methods involve the detection of carbapenemase genes using molecular assays such as polymerase chain reaction (PCR) and whole-genome sequencing (WGS) [5]. These tests vary in performance, cost, turnaround time and labor intensity, and no single test is considered perfect for all situations.

The NG-Test CARBA 5 (CARBA5-LFA) and Carbapenem-resistant K.N.I.V.O. Detection K-Set (KNIVO-LFA) are rapid, qualitative, multiplex lateral flow immunoassays that detect the five major families of carbapenemases. The CARBA5-LFA has been cleared by the US Food and Drug Administration and registered in the European Database on Medical Devices (EUDAMED) as an in vitro diagnostic device, while the KNIVO-LFA has not been FDA-cleared or EUDAMED-registered [6,7]. The CARBA5-LFA has demonstrated excellent sensitivity and specificity, while the performance of the KNIVO-LFA has been shown to be similar [8,9,10,11,12,13,14,15]. However, recent studies have reported false-positive results with both assays, particularly when testing with an overloaded inoculum [16]. The manufacturer’s instructions for the CARBA5-LFA recommend using a loop to touch three colonies of the tested isolate and suspending them in the extraction buffer without specifying the size of the loop or the amount of colony material to be taken in each touch [17]. Meanwhile, the package insert of the KNIVO-LFA suggests collecting the isolate with a loop without mentioning the size of the loop or the number of colonies to be taken [14]. The effect of inoculum size on the performance of these assays remains unclear. In this study, we evaluated the impact of inoculum size on the results of the LFAs with a collection of Enterobacterales that were characterized by whole-genome sequencing.

## 2. Material and Methods

### 2.1. Bacterial Isolates

Testing was conducted in Kwong Wah Hospital, a regional hospital with 1400 beds in Hong Kong. A total of 27 Enterobacterales isolates were randomly selected. These were isolated from clinical specimens (blood, urine and swabs) and sent to the hospital microbiology laboratory from unique patients between November 2021 and November 2023. The isolates included *Morganella morganii*, *Serratia marcescens* complex, *Escherichia coli*, *Klebsiella pneumoniae* complex, *Proteus mirabilis* and *Enterobacter cloacae* complex. Two isolates that had been previously characterized, namely, *E. coli* 7570 carrying NDM-5 and *K. pneumoniae* 7568 carrying OXA-48, were used as positive controls [4]. In addition, *E. coli* ATCC 25922 was used as the negative control. All isolates were cryopreserved at −80 °C after isolation [18].

Before testing, all the isolates were subcultured thrice to ensure pure growth. Firstly, the cryopreserved isolates were streaked onto a chromID CPS Elite (CPS) agar (bioMérieux, Hong Kong) and incubated at 35 ± 2 °C overnight. The colonies on the agar were visually inspected and identified using MALDI-ToF MS with Bruker Microflex LT equipment (Bruker Corporation, Bremen, Germany) and the MBT IVD reference library (MSP-11758, released in August 2022). One colony was then selected from the agar, subcultured onto a second CPS agar and incubated at 35 ± 2 °C overnight. The colonies on the second CPS agar were also inspected and identified with MALDI-ToF MS. A single colony was then picked from the second CPS agar and plated onto a 5% horse blood agar, followed by incubation at 35 ± 2 °C for 18 to 24 h. Finally, colonies on the blood agar were identified with MALDI-ToF MS before testing with the LFAs.

### 2.2. Antimicrobial Susceptibility Testing

The disk diffusion method was used to determine the susceptibility of the isolates to piperacillin-tazobactam, ceftazidime, cefepime, imipenem and meropenem (Thermo Fisher Scientific, Hong Kong, China). The results were interpreted according to CLSI guidelines [18]. Muller Hinton II agar (BD) was used for antimicrobial susceptibility testing (AST).

### 2.3. Inoculum Sizes

**Figure 1:**
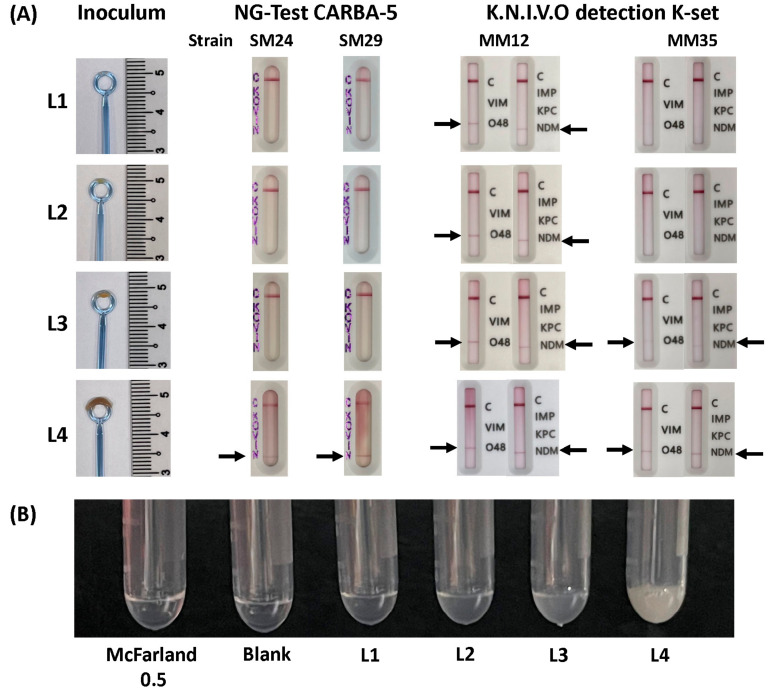
(**A**) Representative images of the inoculum sizes used and corresponding results of the NG-Test CARBA 5 and Carbapenem-resistant K.N.I.V.O. Detection K-Set. The black arrows indicate the false-positive bands encountered in the assays. (**B**) Relative turbidity of the bacterial suspension using different inoculum sizes. The tube on the left contains a bacterial suspension with turbidity equivalent to a 0.5 McFarland standard.

To obtain the inoculum for testing, growth on blood agar was collected using a 10-μL loop. The inoculum size was categorized into four groups (L1–L4) following Tarlton et al. [16]. Inoculum L1 was obtained by touching one area of growth on the blood agar; L2 was obtained by touching three areas of growth; L3 was obtained by touching six areas of growth; and L4 was obtained by touching areas of growth until half of the loop was covered (Figure 1). Additionally, *E. coli* ATCC 25922 was used to prepare bacterial suspensions in saline, which were matched to those obtained by inoculum L1 to L4 in the extraction buffer. The turbidity of the suspensions was measured at a wavelength of 580 nm using a densitometer (DensiCHEK™ Plus instrument, bioMérieux, Marcy-l’Étoile, France). A total of 30 readings were taken for each inoculum, with triplicate readings performed in 10 separate experiments. The McFarland turbidity standards of 0.5, 2.0 and 3.0 corresponded to optical density values ranging from 0.44 to 0.56, 1.85 to 2.15 and 2.79 to 3.21, respectively, for the instrument.

### 2.4. NG-Test CARBA 5

The CARBA5-LFA (NG Biotech, Guipry, France) was performed following the manufacturer’s instructions except for modifications to the inoculum size [17]. Five drops of extraction buffer (150 μL) were added to the provided microtube. Using a 10 μL loop, the growth from the blood agar was transferred to the microtube and resuspended in the extraction buffer. The mixture was vortexed for 30 s. Then, 100 μL of the mixture was added to the sample well of the test cassette using a pipette. The test cassette was incubated at room temperature, and observations were made after 5 min and 15 min. Two members of the study team independently read the results. A negative result was indicated by the presence of only one red line in the control region, while a positive result was indicated by the presence of one red line in the control region and one or more red lines in the test regions, regardless of intensity.

### 2.5. Carbapenem-Resistant K.N.I.V.O. Detection K-Set

The KNIVO-LFA (Genobio Pharmaceutical, Tianjin, China) includes two test cassettes: cassette A detects KPC, NDM and IMP carbapenemases, while cassette B detects VIM and OXA-48-type carbapenemases. The test was performed following the manufacturer’s instructions, except for modifications to the inoculum size [14]. Five drops of sample treatment solution were added to a centrifuge microtube. Using a 10 μL loop, the growth from the blood agar was transferred to the microtube and vortexed for 30 s for homogenization. The mixture (50 μL) was added to each of the test cassettes using a pipette. The cassettes were incubated at room temperature, and observations were made 5 min and 15 min after adding the mixture. Two members of the study team independently read the results. A negative result was indicated by the presence of only one red line in the control region, while a positive result was indicated by the presence of one red line in the control region and one or more red lines in the test regions, regardless of intensity.

### 2.6. Whole-Genome Sequencing and Bioinformatics

Whole-genome sequencing and analysis were performed as per our previous studies [19,20,21]. A DNeasy blood and Tissue kit (Qiagen, Hilden, Germany) was used to extract genomic DNA from cultures. Qualified DNA, checked with a Qubit fluorometer (Invitrogen, CA, USA), was submitted to an Illumina NovaSeq 6000 sequencer at the Novogene Bioinformatics Institute (Beijing, China) [19]. All reads filtered using Trimmomatic v0.39 were assembled using SPAdes v3.15 and evaluated using QUAST v5.0.2 and CheckM v1.2.2 [20,21]. The Genome Taxonomy Database Toolkit (GTDB-Tk v2.3.2) with database release 214 was used to classify all genomes [22]. In the *Serratia* genus, there are 24 validly published species/subspecies. One of them, *Serratia aquatilis*, has no genome sequence. The genomes of the type strains of the other 23 validly published *Serratia* species/subspecies, of *Serratia silvae* (which is not a validly published species) and of strain SM39 (which is an unnamed species designated as clade 1 in a previous publication) were obtained from the NCBI and used as reference strains [23]. The average nucleotide identity (ANI) of the *Serratia* genomes sequenced in this study were calculated with reference strains using FastANI [24]. As reported recently, an ANI of ≥95% is not a suitable cut-off for the designation of *Serratia* species [23]. Therefore, an ANI value ≥97% was used as the threshold for species demarcation. Genome annotations were conducted using Prokka v1.14.5 [25]. Antibiotic resistance genes (ARGs) were identified using Resistance Gene Identifier (RGI) v6.0.3 against the CARD databases 2023 release v3.2.8 with the –include_nudge option [26]. BLASTp was used to search potential hits from all genomes sequenced in the present study against NDM-1 (CAZ39946.1) and OXA-48 (AAP70012.1) with an e-value of 1 and -qcov_hsp_perc of 50 as the cut-off values.

The sequences of the isolates obtained in this study were deposited in the GenBank database under Bioproject PRJNA1100734.

### 2.7. Data Analysis

WGS was used to detect carbapenemase genes in the tested Enterobacterales isolates and acted as the reference method for the current study. The results obtained with the LFAs were compared with the WGS results and were categorized as true negatives if the results were concordantly negative. The detection results of carbapenemases using the LFAs were considered false positives if the corresponding carbapenemase genes were not detected with NGS.

## 3. Results

### 3.1. Bacterial Identification, β-Lactam Susceptibility and Genomic Analysis

The identification and susceptibility of the isolates are summarized in Table 1. MALDI-ToF MS identified the isolates as *M. morganii* (*n* = 10), *S. marcescens* complex (*n* = 10), *E. coli* (*n* = 2), *K. pneumoniae* complex (*n* = 2), *P. mirabilis* (*n* = 2) and *E. cloacae* complex (*n* = 1). WGS resolved the 10 *S. marcescens* complex isolates as *Serratia* clade 1 (*n* = 3), *S. bockelmannii* (*n* = 2), *S. nematodiphila* (*n* = 1), *S. nevei* (*n* = 1), and *S. ureilytica* (*n* = 1) (Supplementary Materials, Table S1). The *E. cloacae* complex isolate was resolved as *E. roggenkampii* (*n* = 1). For the remaining isolates, identifications obtained by WGS were the same as those obtained by MALDI-ToF MS. All the isolates were susceptible to meropenem, while susceptibilities to piperacillin-tazobactam, ceftazidime, cefepime and imipenem were variable.

In all the isolates, no carbapenemase gene was detected in the genomic analysis (Table 1). Several class C (*bla*_SRT_, *bla*_SST_, *bla*_MIR_) and class A (*bla*_TEM-1b_, *bla*_SHV-110_) β-lactamase genes were detected in the isolates. However, the similarities of these β-lactamase sequences with *bla*_NDM-1_ and *bla*_OXA-48_ were very low (Supplementary Materials, Table S2). The presence of NDM and OXA-48-like protein sequences was further explored using BLASTp with loose parameters. The hits involved short sequences with low-percentage identities. There were no hits at ≥50% coverage and ≥50% identity (Supplementary Materials, Table S2).

### 3.2. Testing with NG-Test CARBA 5 and Carbapenem-Resistant K.N.I.V.O. Detection K-Set

The bacterial suspensions of inoculum sizes L1, L2 and L3 exhibited turbidity levels close to McFarland standards of 0.5, 2.0 and 3.0, respectively (Supplementary Materials, Table S3). The optical density value of L4 was above the upper limit for the densitometer, indicating that its turbidity was at least a McFarland standard of 4.0.

Overall, very high proportions of the *Serratia* and *Morganella* isolates yielded false-positive bands in the two LFA tests (Table 2). This involved NDM in CARBA5-LFA, and both NDM and OXA-48 in the KNIVO-LFA. In both tests, the positive and negative controls yielded the expected results at all inoculum sizes.

For the CARBA5-LFA, the flow of the assay was smooth at inoculum sizes L1 to L3, and the control band could be seen 5 min after the addition of the mixture to the sample well. The results observed at the 5th min were the same as those observed at the 15th min. However, for inoculum L4, the flow of the assay was much slower. At 5 min after the addition of the mixture, the flows for some assays were not complete, but all results could be read at the 15th min. None of the 27 isolates tested positive for carbapenemases at inoculum sizes L1 to L3. However, eight *Serratia* isolates and six *M. morganii* isolates showed false-positive NDM results at inoculum L4. The CARBA5-LFA test was negative for the other isolates at all inoculum sizes, which is consistent with the WGS results. The false-positive bands varied in intensity, with most appearing fainter than the control band (Figure 1). To assess the reproducibility of the results, all *Serratia* and *M. morganii* isolates were retested with a different lot of the CARBA5-LFA tests and on a different date. For the *Serratia* isolates, the test results were concordant in both runs, with no false-positive results using inoculum sizes L1 to L3, but false-positive results for NDM with inoculum L4 for all eight isolates mentioned. Five out of six of the *Morganella* isolates (MM11, MM12, MM35, MM56 and MM58) showed false-positive NDM results with inoculum L4 in the repeat experiment, which were largely similar to the initial testing results (Supplementary Materials, Table S4).

The KNIVO-LFA showed a faster flow compared to the CARBA5-LFA. At all tested inoculum sizes, the assays were complete by 5 min after adding the mixture, with a clearly visible control band. However, the false-positive bands took a longer time to appear compared to the control bands. Most false-positive bands were not visible at 5 min but could be observed at 15 min. The false-positive bands also appeared fainter compared to the control bands. At inoculum L4, three *Serratia* isolates (SM01, SM24, SM35) showed false-positive results for NDM and OXA-48-like carbapenemases, while one (SM09) was a false positive for NDM only (Table 2). All *M. morganii* isolates demonstrated false-positive results for NDM and/or OXA-48-like enzymes with the assay at L4. The results for L1 to L3 were variable. A *K. pneumoniae* isolate was false-positive for NDM and/or OXA-48-like enzymes with inoculum sizes L3 and L4. Upon retesting, results consistent with the initial findings were obtained (Supplementary Materials, Table S4).

## 4. Discussion

This study demonstrates the frequent occurrence of inoculum-dependent, false-positive results in *Serratia* and *Morganella* species when using two LFAs. The KNIVO-LFA exhibited false-positive results in two *M. morganii* isolates even with a light inoculum, suggesting that these false results were not solely due to inoculum size. It is important to note that multiple species from three bacterial genera were involved. In LFAs, it is well known that false-positive results can be caused by the inadequate optimization of reaction buffers, nonspecific protein–conjugate interactions and cross-reactivity [27,28]. Our findings corroborate previous studies showing that the false-positive results mostly involved the NDM band for the CARBA5-LFA and both the NDM and OXA-48-like bands for the KNIVO-LFA, with the intensities of the false-positive bands being fainter than the control bands (Table 3). It is important to highlight that a diverse range of inoculum sizes, varying from one to seven colonies, have been utilized in studies that demonstrated the occurrence of false-positive results (Table 3). While the difference in inoculum sizes may have influenced the test results, false-positive results have been reported even when a 1 μL loop was used to obtain the isolate for testing [29]. Further investigations would be needed to determine the underlying cause.

In routine service, it is probable that most operators would utilize inoculum sizes similar to those of L1 to L3 when conducting LFAs for carbapenemase detection. However, due to the mucoid nature and substantial variation in colony sizes in Enterobacterales, there may be inadvertent use of larger inoculum sizes if adequate care is not taken during test preparation in a busy laboratory. Misidentifying clinical isolates as carbapenemase producers can result in the unnecessary escalation of antimicrobial therapy and infection control measures, which can have negative consequences for patients. Improvements in instructions from the manufacturer are crucial to prevent the use of overloaded inocula in LFAs. The product insert of the KNIVO-LFA suggests that users suspend the bacterial colony in the sample treatment solution with a loop, without specifying the size of the loop or the number of colonies to be used, which may result in significant variations in inoculum sizes among different operators [14]. The package insert for the CARBA5-LFA, released in 2022, states that three colonies should be touched with a loop, without specifying the size of the loop, but it was revised in the 2023 version to state that a 1 μL loop should be filled by touching (but not collecting) three colonies [17,34]. The inoculum size suggested in the 2023 revision is equivalent to the L1 inoculum in our study. Including the suggested loop size and specifying that colonies should be touched but not collected would standardize inoculum sizes among different operators and consequently reduce the possibility of false-positive test results. Alternatively, manufacturers could quantify the inoculum size using the turbidity of the resultant bacterial suspension (such as by providing the range of acceptable McFarland standards for the suspension) to minimize subjectivity and inter-operator variability.

A limitation of this study is the small sample size, with a predominant focus on the *Serratia* and *M. morganii* species. However, the use of WGS to verify the absence of carbapenemase genes and to confirm bacterial species is a strength of this study. To further evaluate the influence of inoculum size on the performance of LFAs in other Enterobacterales members, additional investigations are needed.

In summary, the present study showed that the occurrence of false-positive NDM and OXA-48-like results in *Serratia* and *Morganella* species is dependent on the inoculum size when using two commercially available LFAs. These results underscore the importance of providing clearer instructions regarding the optimal inoculum size and the inclusion of this requirement for regulatory approval.

## Figures and Tables

**Table 1:** Identification and susceptibility of isolates (*n* = 27) in this study.

Strain	Bacterial Identification by		Resistance Pattern ^a^	Presence of β-Lactamase Gene(s) by WGS		
	MALDI-ToF MS	WGS		*bla*_NDM_	*bla*_OXA-48_	Others
SM01	*Serratia marcescens*	*Serratia nevei*	SSSSS	-	-	*bla*_SRT-2_
SM04	*Serratia marcescens*	*Serratia* clade 1	SSSSS	-	-	*bla*_SRT-2_
SM09	*Serratia marcescens*	*Serratia bockelmannii*	SSSSS	-	-	*bla*_SRT-2_
SM21	*Serratia marcescens*	*Serratia ureilytica*	SSSSS	-	-	*bla*_SST-1_
SM24	*Serratia marcescens*	*Serratia nevei*	SSSSS	-	-	*bla*_SRT-2_
SM29	*Serratia marcescens*	*Serratia* clade 1	ISSSS	-	-	*bla*_SRT-2_
SM35	*Serratia marcescens*	*Serratia nevei*	SSSSS	-	-	*bla*_SRT-2_
SM43	*Serratia marcescens*	*Serratia nematodiphila*	SSSSS	-	-	*bla*_SST-1_
SM59	*Serratia marcescens*	*Serratia* clade 1	SSSSS	-	-	*bla*_SRT-2_
SM66	*Serratia marcescens*	*Serratia bockelmannii*	SSSSS	-	-	*bla*_SRT-1_
MM04	*Morganella morganii*	*Morganella morganii*	IRSIS	-	-	*bla*_DHA-17_
MM11	*Morganella morganii*	*Morganella morganii*	SSSIS	-	-	*bla*_DHA-2_
MM12	*Morganella morganii*	*Morganella morganii*	SSSIS	-	-	*bla*_DHA-4_
MM13	*Morganella morganii*	*Morganella morganii*	SSSSS	-	-	*bla*_DHA-17_
MM35	*Morganella morganii*	*Morganella morganii*	SSSIS	-	-	*bla*_DHA-13_
MM55	*Morganella morganii*	*Morganella morganii*	SSSSS	-	-	*bla*_DHA-17_
MM56	*Morganella morganii*	*Morganella morganii*	SSSIS	-	-	*bla*_DHA-14_
MM58	*Morganella morganii*	*Morganella morganii*	SSSSS	-	-	*bla*_DHA-4_
MM64	*Morganella morganii*	*Morganella morganii*	SSSSS	-	-	*bla*_DHA-1_
MM65	*Morganella morganii*	*Morganella morganii*	SSSIS	-	-	*bla*_DHA-14_
EC04	*Escherichia coli*	*Escherichia coli*	SSSSS	-	-	-
EC25	*Escherichia coli*	*Escherichia coli*	SSSSS	-	-	*bla*_TEM-1B_
KP15	*Klebsiella pneumoniae*	*Klebsiella pneumoniae*	SSSSS	-	-	*bla*_SHV-1_
KP25	*Klebsiella pneumoniae*	*Klebsiella pneumoniae*	SSSSS	-	-	*bla*_SHV-110_
PM23	*Proteus mirabilis*	*Proteus mirabilis*	SSSSS	-	-	-
PM82	*Proteus mirabilis*	*Proteus mirabilis*	SSSSS	-	-	-
EL06	*Enterobacter cloacae*	*Enterobacter roggenkampii*	SISSS	-	-	*bla*_MIR-1_

^a^ Resistance pattern for piperacillin-tazobactam, ceftazidime, cefepime, imipenem, meropenem. “-”, not detected; S, sensitive; I, intermediate (susceptible dose-dependent for piperacillin-tazobactam and cefepime); R, resistant; WGS, whole-genome sequencing.

**Table 2:** Results of testing with NG-Test CARBA 5 and Carbapenem-resistant K.N.I.V.O. Detection K-Set.

Strain	Genomic Species	CARBA 5 Results by ^a,b,c^				K.N.I.V.O. Detection K-Set Results by ^b,c^			
		L1	L2	L3	L4	L1	L2	L3	L4
SM01	*Serratia nevei*	-	-	-	N (++)	-	-	-	N (w), O (w)
SM04	*Serratia* clade 1	-	-	-	N (+)	-	-	-	-
SM09	*Serratia bockelmannii*	-	-	-	N (++)	-	-	-	N (w)
SM21	*Serratia ureilytica*	-	-	-	N (+)	-	-	-	-
SM24	*Serratia nevei*	-	-	-	N (+)	-	-	-	N (w), O (w)
SM29	*Serratia* clade 1	-	-	-	N (++)	-	-	-	-
SM35	*Serratia nevei*	-	-	-	N (+)	-	-	-	N (++), O (++)
SM43	*Serratia nematodiphila*	-	-	-	-	-	-	-	-
SM59	*Serratia* clade 1	-	-	-	N (+)	-	-	-	-
SM66	*Serratia bockelmannii*	-	-	-	-	-	-	-	-
MM04	*Morganella morganii*	-	-	-	-	-	-	-	N (w), O (w)
MM11	*Morganella morganii*	-	-	-	N (+)	-	-	N (w), O (w)	N (+), O (+)
MM12	*Morganella morganii*	-	-	-	N (w)	N (++), O (++)	N (++), O (++)	N (++), O (++)	N (++), O (++)
MM13	*Morganella morganii*	-	-	-	-	-	-	-	N (w)
MM35	*Morganella morganii*	-	-	-	N (w)	-	-	N (w), O (w)	N (++), O (++)
MM55	*Morganella morganii*	-	-	-	-	-	-	O (w)	N (+), O (+)
MM56	*Morganella morganii*	-	-	-	N (+)	-	-	-	N (w)
MM58	*Morganella morganii*	-	-	-	N (w)	-	-	-	N (w)
MM64	*Morganella morganii*	-	-	-	-	N (+), O (+)	N (++), O (++)	N (++), O (++)	N (++), O (++)
MM65	*Morganella morganii*	-	-	-	N (+)	-	-	-	N (w)
EC04	*Escherichia coli*	-	-	-	-	-	-	-	-
EC25	*Escherichia coli*	-	-	-	-	-	-	-	-
KP15	*Klebsiella pneumoniae*	-	-	-	-	-	-	-	-
KP25	*Klebsiella pneumoniae*	-	-	-	-	-	-	N (+), O (++)	O (w)
PM23	*Proteus mirabilis*	-	-	-	-	-	-	-	-
PM82	*Proteus mirabilis*	-	-	-	-	-	-	-	-
EL06	*Enterobacter roggenkampii*	-	-	-	-	-	-	-	-

^a^ L1 to L4 represent the four different inoculum sizes. ^b^ -, negative; N, NDM; O, OXA-48. ^c^ The intensity of the false-positive bands: ++, similar intensity to the control band; +, the band can be readily read but less intense than the control band; w, faint band.

**Table 3:** Summary of the literature on false-positive carbapenemase results in Gram-negative bacteria detected by lateral flow assays.

Name of Lateral Flow Assays (Reference)	Organisms (Number with False-Positive Result/Number Tested)	Size of Bacterial Inoculum Used for Test Performance	False-Positive Target (Band Intensity)	Reference Method
NG-Test CARBA 5 [11]	*Klebsiella pneumoniae* (1/214)	Followed manufacturer’s instructions ^a^; loop size not described	NDM (nd)	PCR and sequencing
NG-Test CARBA 5 [29]	*Enterobacter cloacae* (1/299)	Followed manufacturer’s instructions ^a^; 1 μL loop was used	NDM (nd)	WGS
NG-Test CARBA 5 [16]	*Morganella morganii* (3/11). False-positive results only observed with extra heavy inoculum. Same false-positive results obtained upon retesting of the 3 isolates.	Multiple inoculum sizes were tested using 10 μL loops. Light inoculum: touch 1 area of growth with the loop; standard inoculum: touch 3 areas of growth with the loop; heavy inoculum: touch 6 areas of growth; extra heavy inoculum: fill half of a 10 μL loop	NDM (w) only seen with extra heavy inoculum	PCR and/or WGS
NG-Test CARBA 5 [30]	*Serratia marcescens* (1/197)	One fresh colony was suspended in 5 drops of extraction buffer	OXA-48 (w)	PCR and/or WGS
NG-Test CARBA 5 [31]	*Acinetobacter baumannii* (15/24)	Followed manufacturer’s instruction ^a^; loop size not described	IMP (w)	WGS
NG-Test CARBA 5 [32]	*Acinetobacter baumannii* (93/97 for the first lot, 61/97 for the second lot)	Touched 6–7 colonies for the first lot and 3–4 colonies for the second lot using a 1 μL loop, and then suspended in 5 drops extraction buffer	IMP (w to +)	WGS
NG-Test CARBA 5 [12]	Enterobacterales, *Acinetobacter* spp. and *Pseudomonas* spp. (2/197)	One colony of overnight growth harvested from Columbia blood agar plate was suspended in 5 drops of extraction buffer; loop size not described	VIM (*n* = 1, nd), KPC (*n* = 1, nd) ^b^	PCR and/or WGS
Carbapenem-resistant K.N.I.V.O. Detection K-Set [33]	*Stenotrophomonas maltophilia* (1/1)	Not described	NDM (+), OXA-48 (+)	PCR

^a^ Touch 3 colonies with a loop and then suspend it in a microtube containing 5 drops (150 μL) of extraction buffer. ^b^ The species of the two isolates that gave a false-positive VIM band, and a KPC band was not reported. True-negative results were obtained for both isolates on repeat testing using the NG-Test CARBA 5. The intensity of the false-positive bands: +, the band can be readily read but less intense than the control band; w, faint band; nd, not described.

## Data Availability

All relevant data have been included in the manuscript.

## Supplementary Materials

The following supporting information can be downloaded at: https://www.mdpi.com/article/10.3390/diagnostics14121274/s1, Table S1: Identification of *Serratia* species by Average Nucleotide Identity (FastANI) percentages; Table S2: BLASTP analysis of isolates in this study against reference sequence of NDM-1 (CAZ39946.1) and OXA-48 (AAP70012.1); Table S3: Optical densitiy measurement for inoculum L1 to L3 using *Escherichia coli* ATCC 25922; Table S4: Results of testing by NG-Test CARBA 5 and Carbapenem-resistant K.N.I.V.O. Detection K-Set results.
